# Quantum Dot- Conjugated Anti-GRP78 scFv Inhibits Cancer Growth in Mice

**DOI:** 10.3390/molecules17010796

**Published:** 2012-01-16

**Authors:** Weiming Xu, Lizhi Liu, Nicola J. Brown, Sven Christian, David Hornby

**Affiliations:** 1 Department of Molecular Biology and Biotechnology, The Krebs Institute, The University of Sheffield, S10 2TN, UK; Email: Lizhi.liu@sheffield.ac.uk (L.L.); d.hornby@sheffield.ac.uk (D.H.); 2 Academic Surgical Oncology Unit, Department of Oncology, Faculty of Medicine, Dentistry and Health, University of Sheffield, S10 2RX, UK; Email: n.j.brown@sheffield.ac.uk; 3 Bayer Pharma AG, BSP-GDD-GTR-TD-TR2W, Aprather Weg 18a, 2096 Wuppertal, Germany; Email: sven.christian@bayer.com

**Keywords:** Quantum Dot, scFv, GRP78, nanoparticle, AKT, breast cancer

## Abstract

Semiconductor quantum dots (Qdots) have recently been shown to offer significant advantages over conventional fluorescent probes to image and study biological processes. The stability and low toxicity of QDs are well suited for biological applications. Despite this, the potential of Qdots remains limited owing to the inefficiency of existing delivery methods. By conjugating Qdots with small antibody fragments targeting membrane-bound proteins, such as GRP78, we demonstrate here that the Quantum dot- Anti-GRP78 scFv (Qdot-GRP78) retains its immunospecificity and its distribution can be monitored by visualization of multi-color fluorescence imaging both *in vitro* and *in vivo*. Moreover we demonstrate here for the first time that Qdot-GRP78 scFv bioconjugates can be efficiently internalized by cancer cells, thus upregulate phophosphate-AKT-ser473 and possess biological anti-tumour activity as shown by inhibition of breast cancer growth in a xenograft model. This suggests that nanocarrier-conjugated scFvs can be used as a therapeutic antibody for cancer treatment.

## 1. Introduction

Quantum dots (Qdots) are semiconductor nanocrystals 2 to 10 nm in diameter (200–10,000 atoms), which emit fluorescence depending on their particle size [1]. Compared with conventional bioorganic fluorophores, Qdots release more than 20 times the fluorescence intensity and are resistant to photo-bleaching upon excitation. Recently, Qdots have emerged as superior new dyes with a large number of potential applications in fluoroimmunoassays and biological imaging [2]. For example, Qdots conjugated to trastuzumab (Herceptin) have been used for *in vivo* real-time tracking in breast cancer cells [3]. However, conjugating full-length monoclonal antibodies directly to Qdots is a relatively difficult process. In contrast, single chain antibody fragments (scFv) have a relatively small size (approximately 30 kDa) and are generally amenable to be genetically and structurally manipulated [4], and therefore have advantages over monoclonal antibodies as carriers of radionuclei, drugs and nanobeads. Recently, a human tumor-targeting scFv-modified nanoparticle was used to deliver siRNA and miRNA to lung cancer cells in a syngeneic murine model [5].

In this report, we selected GRP78 as our molecular target to assess the function of a Qdot-labeled scFv antibody. GRP78 is an ER resident protein which plays a crucial role in cancer cell proliferation and angiogenesis [6]. Recent research has shown that various cancers [7], including breast and prostate cancer [8], express membrane-associated GRP78. Previously we reported that nitric oxide induced coupling of mitochondrial respiration to ER-stress, resulting in increased GRP78 expression [9]. Furthermore, we have previously targeted membrane-bound GRP78 by subtractive screening of a single chain variable fragment (scFv) library and have successfully isolated a panel of scFvs specific for GRP78 [10]. In this work, we describe proof-of-concept studies investigating the therapeutic potential of a quantum dot-nanobead labeled scFv-GRP78-H19 antibody in a preclinical xenograft nude mouse model and demonstrate that it performs biological functions *in vitro* in tissue culture and *in vivo* in a xenograft model in mice.

## 2. Results and Discussion

### 2.1. Conjugation of an Anti-GRP78 scFv with Quantum Dot-625 Nanoparticle

**Figure 1:**
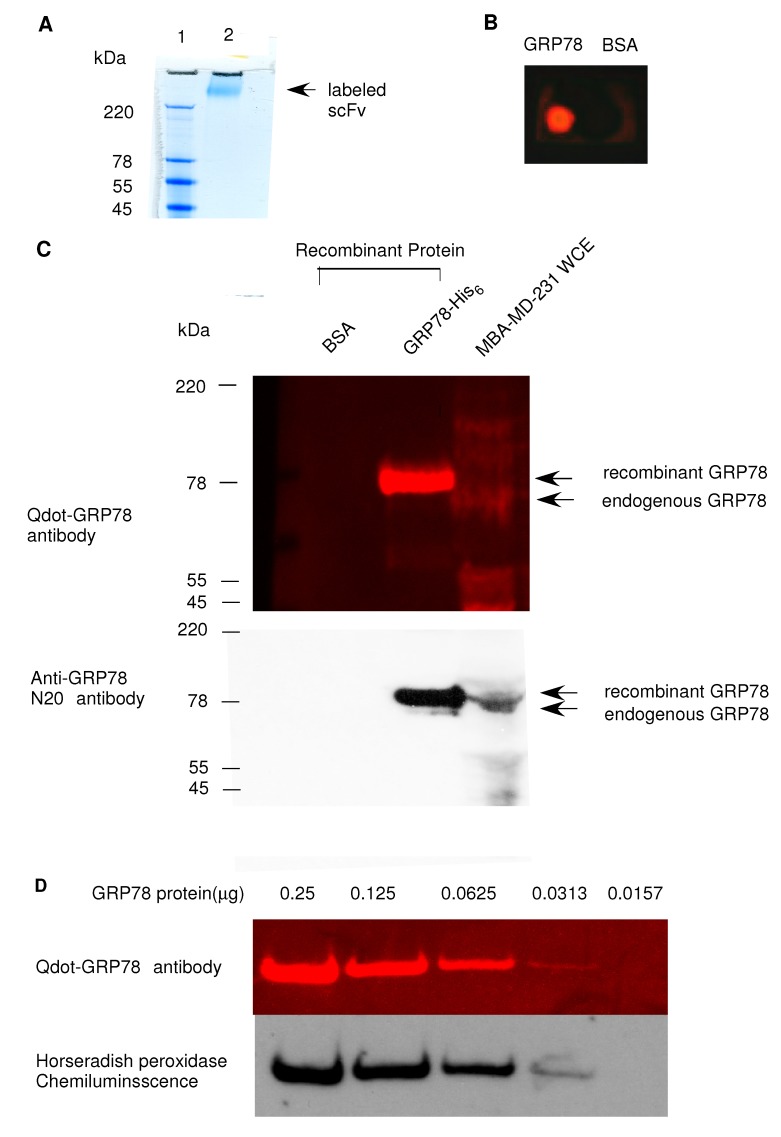
scFv-Grp78-H19 antibody was conjugated to a Qdot-625. (**A**) Coomassie blue-stained SDS-PAGE gel showing the Qdot labeled scFv; (**B**) Dot blot analysis of GRP78 protein binding by the Qdot625 labeled scFv; (**C**) Western blot analysis of GRP78 protein binding with Qdot625-GRP78 antibody with recombinant protein or whole cell extract (WCE) (upper panel). The same blot was hybridized with a goat-anti-GRP78 polyclonal antibody [N20, detected by horseradish peroxidase chemiluminescence (lower panel)]; (**D**) GRP78 protein was diluted in electrophoresis buffer and serial dilutions were then prepared and the western blot was probed with either anti-GRP78-Qdot625 scFv antibody (detected by UV illumination) or anti-GRP78-C20 polyclonal antibody (detected by horseradish peroxidase chemiluminescence).

We have conjugated scFv-GRP78-H19 [10] to quantum dot-625 (Invitrogen) with an antibody conjugation kit according to the manufacturer’s instructions. The Q-dot- scFv-GRP78-H19 Conjugates(Qdot-GRP78) were then concentrated by ultrafiltration and purified by size exclusion chromatography with a molecular weight of over 220 kDa being observed (Figure 1A). We have used both dot-blot and Western blot to test the specificity of the antibody to GRP78. In the dot-blot experiments, BSA and murine GRP78 (200 μg/mL) were spotted on a nitrocellulose filter and probed with 10 nM Qdot-GRP78 antibody overnight with PBSA solution in 1% nonspecific rabbit serum. We observed a very strong intensity of red dots under UV transilluminator (UVP gel document system, Cambridge, UK) on the GRP78 protein spot (Figure 1B); no signal was detected on the control BSA spot.

We next performed a polyacrylamide gel electrophoresis on recombinant GRP78 protein and whole cell extracts (WCE) of human breast cancer MDA-MB-231/GFP cell samples. The gel was directly stained with 10 nM Qot-GRP78 antibody overnight. Again, we observed a very strong intensity red band in the lane which contained recombinant GRP78 protein under UV illumination. No signal was detected in the BSA lane (Figure 1C, upper panel). We also detected the endogenous GRP78 signal from whole cell extracts of MDA-MB-231/GFP cells. It should be noted that the endogenous GRP78 band migrates more slowly than the recombinant GRP78 bands which contains the 6 HIS-tag. To corroborate the gel staining results, we made the Western blot with the same gel. As shown on the lower panel of Figure 1C, the GRP78 band from the whole cell extract migrated fairly slower than that of the recombinant GRP78. To compare the sensitivity of the Qdot probe with a conventional Western blot, GRP78 protein was diluted in electrophoresis buffer and subjected to Western blot analysis with either Qdot-GRP78 antibody or the goat polyclonal anti-GRP78 antibody with conventional secondary antibody: HRP-conjugated Anti-Goat IgG (Figure 1D). Both methods detected protein orders ranging from 0.25 μg to 0.03 μg, with Qdot antibodies only requiring a single step, while conventional ECL detection needs multiple steps and enzymatic amplification.

**Figure 2:**
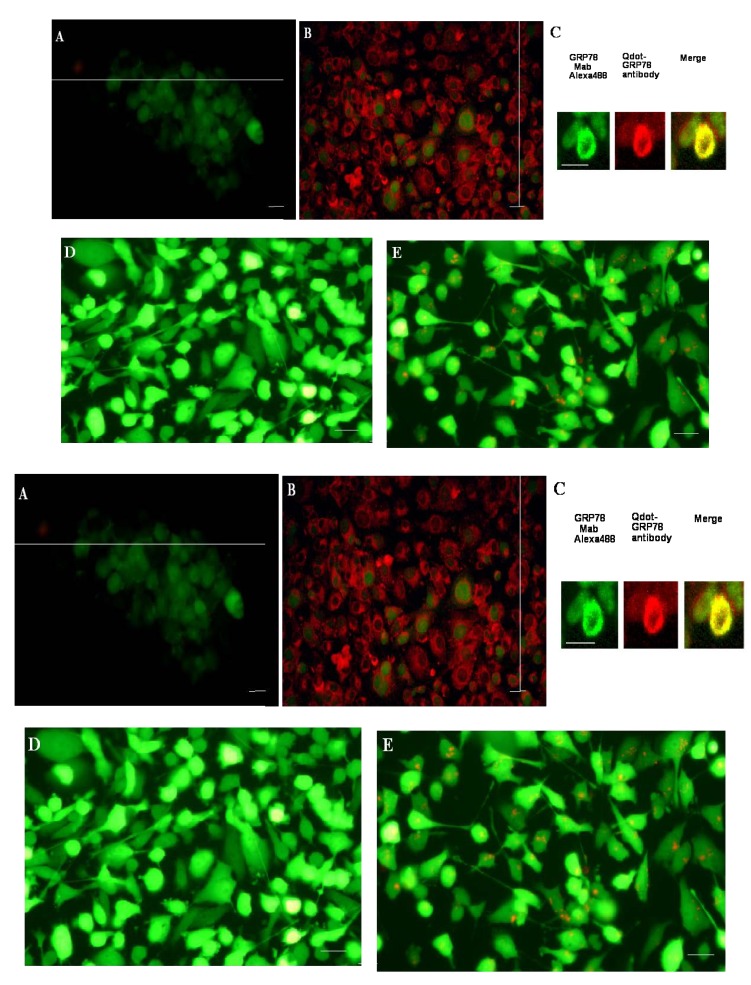
Immunohistochemistry of Qdot-GRP78 antibody in MDA-MB-231/GFP breast cells and LNCaP prostate cancer cells. (**A**) and (**B**) Fluorescence images of cells staining with Qdot625-GRP78 antibody. (**A**) control cells staining with unlabeled nanobeads; (**B**) MDA-MB-231/GFP cells staining with Qdot-GRP78 antibody; (**C**) Scanned confocal microscopy imaging in LNCaP prostate cancer cell, stained with anti-GRP78 mouse monoclonal antibody with secondary Goat anti-mouse IgG labeled with Alexa488(green)and Qdot-GRP78(red dots) and overlapped with two probes (yellow); (**D**) and (**E**) Internalization of Qdot-GRP78 antibody by MDA-MB-231/GFP cells. Cells were incubated with unlabelled nanobeads. (**D**) or Qdot-GRP78 antibody; (**E**) at 37 °C for 16 h. Cells were then washed with PBS and analyzed by fluorescence microscopy; (**F**) Scanned confocal microscopy imaging in MDA-MB-231/GFP cell (green), treated with control unlabelled beads; (**G**) Qdot-GRP78(red dots) was detected inside MDA-MB-231/GFP cell; (**H**) 3D reconstruction of confocal Z stack with 0.8-μM, GFP cell showing in green channel, while Qdot-GRP78 showing in red channel. Scale bar represents 20 μm; (**I**) Western blot analysis of GRP78 protein in Qdot-GRP78 antibody treated cells. Cells either treated with control (unlabelled Qdot) or Qdot-labeled antibody. Phosphorylated Akt-ser473 protein was detected by an anti-anti-Akt-se473 antibody and Pan-Akt antibody was used as a loading control. The western blot represents three independent experiments. (**J**) MDA-MB-231/GFP cells were incubated with various concentrations of Qot-GRP78 antibody for 24h. Apoptotic nuclei or nuclear DNA strand breaks were visualized using Hoechst DNA dye H33342 (10). A minimum of 200 cells were counted in each sample and condensed or fragmented nuclei were expressed as a percentage of the total number of nuclei. Values are presented as mean ± S.D. of three determinations. * indicates significant difference (*p* < 0.05) between none treatment cells and antibody treatment cells.

**Figure 3:**
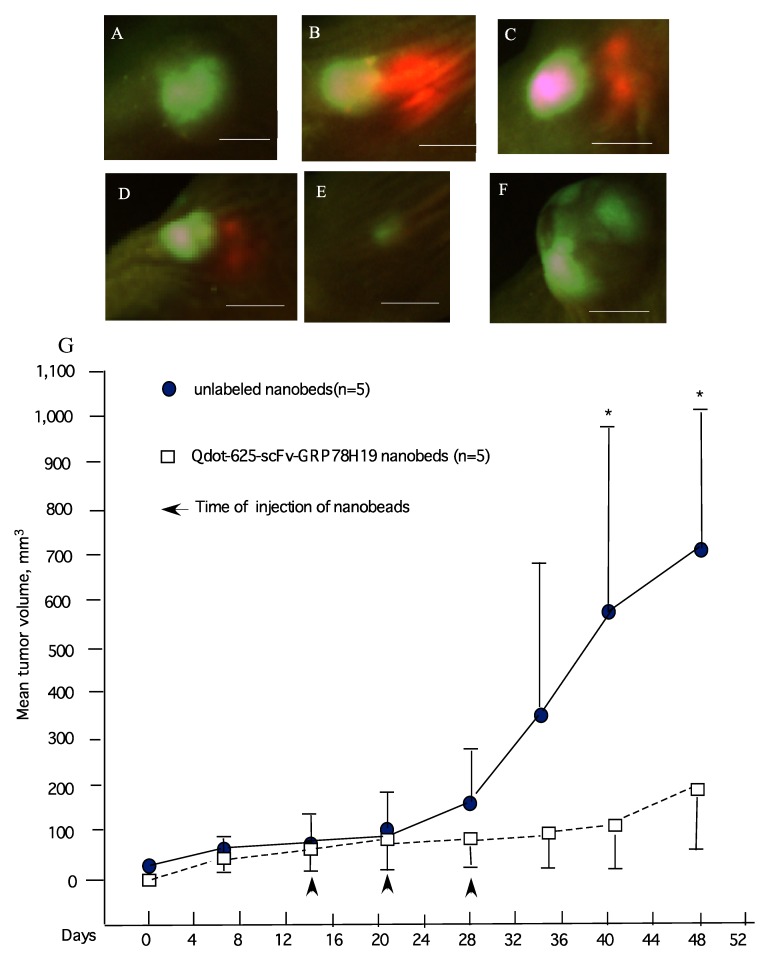
Qdot-GRP78 antibody-conjugates inhibits breast cancer growth. Two-color fluorescence imaging of effect of the injected antibody(red) and GFP tumor(green). (**A**) before injection; (**B**) two days after injection; (**C**) five days after injection; (**D**) two weeks after injection; (**E**) five weeks after injection; (**F**) five weeks after unlabeled nanobeads only injection; (**G**) Total 2 × 10^6^ tumor cells were injected subcutaneously into each Balb/c nu/nu mouse. Each experiment used five female mice. In the treatment group, the Qdot-GRP78 antibody was intratumorally injected into pre-established tumors (on the second week) (white square). Then injected at weekly interval for three weeks. In control groups, mice received unlabelled nanobeads alone (black circle. * *p* < 0.05, two-tailed Student’s *t* test. Scale bar represents 5 mm.

We then performed immunocytochemistry on breast cancer MDA-MB-231/GFP cells using Qdot-GRP78 antibody. As shown in Figure 2B, the antibody stained both the membrane and ER in the breast cancer cells, with no specific staining in the control cells in which the unlabeled Qdot-625 nanobeads were used (Figure 2A). To further confirm GRP78 staining of the antibody, we colocalized our Qdot antibody with an anti-GRP78 mouse monoclonal antibody in the LNCaP prostate cancer cell (Figure 2C). Mouse anti-GRP78 can be detected with fluorescence Alexa488 labeled secondary Goat anti-mouse IgG (green), while our Qdot antibody can be detected by red fluorescence. Scan confocal microscopy imaging in LNCaP prostate cancer cell shows the overlapping between the two antibodies (yellow). In order to determine if Qdot-GRP78 antibody was capable of being internalized, the labeled antibody was incubated with MDA-MB-231/GFP cells for 24 h, and after washing with PBS cells were directly examined using a fluorescence microscope. Internalisation of the Qdot-GRP78 antibody (red dots) into the MDA-MB-231/GFP cells was observed in more than 30% cells (Figure 2E), while no specific staining was observed in unlabeled Qdot-625 nanobeads (Figure 2D), indicating efficient internalization of the Qdot-GRP78 antibody. Confocal microscopy was used to examine the details of the Qdot internalization. While no specific red fluorescence was detected in unlabeled Qdot-625 treated samples (Figure 3F), the Qdot-GRP78 (red, Figure 2G) was internalized in MDA-MB-231/GFP cells. Figure 2H shows the same image of the 2G, with 3D reconstructed scanning confocal images. In Western blot analysis (Figure 2I), we found that after treating cells with Qdot-GRP78 antibody for 16 h, the phosphorylated AKT-serine 473 expression level has significantly increased in comparison with that of unlabeled Qdot-625 treated samples, while pan-AKT level remains the same. To detect cell apoptosis, MDA-MB-231/GFP cells were incubated with various concentrations of Qot-GRP78 antibody for 24 h. The cells were then stained with Hoechst DNA dye H33342 (10). We found that there was a dose-dependent increase apoptosis in Qdot-GRP78 treated cells (Figure 2J).

### 2.2. QD scFv Bioconjugates Inhibit Breast Cancer Growth in a Xenograft Model

The advantage of the Qdot-conjugated antibody is that it can be easily used in multi-color experiments, where we can detect and monitor the antibody/antigen interactions *in vivo*. In this study we used GFP labeled breast cancer MDA-MB-231/GFP cells, subcutaneously transplanted into the nude mice. When the tumor reached a volume of approximately 50–100 mm^3^ within a two to three week period, Qdot-625-scFv-GRP78 (80 μL containing 80 nM antibody) was intratumorally injected into the pre-established GFP labeled tumors (Figure 3). Tumor growth and antibody distribution can be monitored by visualization of multi-color fluorescence imaging (green representing tumor, red representing antibody) with a single excitation source (470 nm excitation filter) and single emission filter (515 nm viewing filter; LightTools Research, Encinitas, CA, USA) (Figure 3A–E). A total of three injections were carried out at weekly intervals. Treatment of established tumors with the Qdot-GRP78 scFv antibody-conjugates significantly inhibits the breast tumour growth (191.1 ± 150 mm^3^, Figure 3E,G) in comparison with the samples treated only with unlabeled nanobeds (717 ± 335 mm^3^, Figure 3F,G.) seven weeks following implantation) which represents a 74% reduction in tumor volume (Students *t* test, *p* < 0.05).

**Figure 4:**
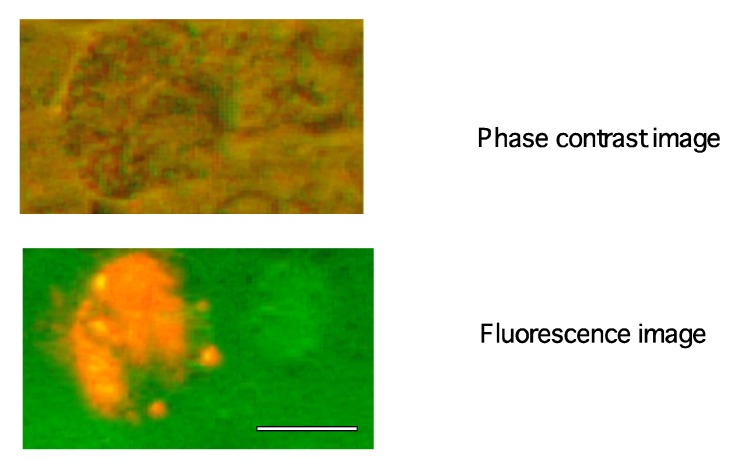
Qdot-GRP78 antibody detected in the tumor samples. The red fluorescence of Qdot-GRP78 antibody was detected in some of the cells inside tumor. Scale bar represents 20 μm.

At the end of seven weeks, three mice treated with Qdot-625-scFv-GRP78 antibody still showed a measurable tumor mass. The red fluorescence of scFv-GRP78 was detected in some of the cells inside tumors under fluorescence microscope (Figure 4). It should be noted that we were unable to detect any red fluorescence cells in other organs (kidney, heart, liver, spleen and lung, Supplementary Figure S1). 

### 2.3. Discussion

Recent groundbreaking work has established that GRP78 protein, a master switch in ER stress, plays a crucial role in cancer cell proliferation, angiogenesis and chemotherapy drug resistance [7]. Cell surface Grp78 expression has been detected in many different cancers, such as breast, liver, and prostate cancer [7,11]. Previously we reported that nitric oxide induced coupling of mitochondrial respiration to ER-stress, inducing GRP78 expression and subsequent regulation [9]. Furthermore, we have targeted membrane-bound GRP78 by subtractive screening of a single chain variable fragment (scFv) library and successfully isolated a panel of scFv specific for GRP78 [10]. The isolated scFv-GRP78 detected breast cancer cell surface Grp78 (Figure S2A and membrane locations are indicated by arrows) on the breast carcinoma cells (Tx N1, stage II), as well as cancer cell surfaces in metastatic lymph nodes (Figure S2C,) in a high- density paraffin-embedded breast cancer microarray (Clinomics Biosciences, Inc). The epitope region of the antibody is on the N-terminus of GRP78 [10]. Recent research has shown that both the C-terminus and N-terminus of GRP78 play important roles in ER-stress response signalling pathways. Misra *et al.* demonstrated that antibodies directed against the COOH-terminal domain of GRP78 up-regulated the tumor suppressor protein p53 [12], while several other studies have shown that antibody directed against the NH (2)-terminal domain regulated the prostate apoptosis response-4(par-4) signaling pathway [13] and Cripto pathways [14]. Burikhanov *et al.* showed that antibody against the N-terminus of GRP78 neutralized cellular apoptosis induced by the *par-4*, a cancer cell-selective pro-apoptotic protein [13]. Furthermore, neutralizing antibody against the N-terminus of GRP78 inhibited oncogenic *Cripto* signaling via MAPK/PI3K and Smad2/3 pathways [14].

**Figure 5:**
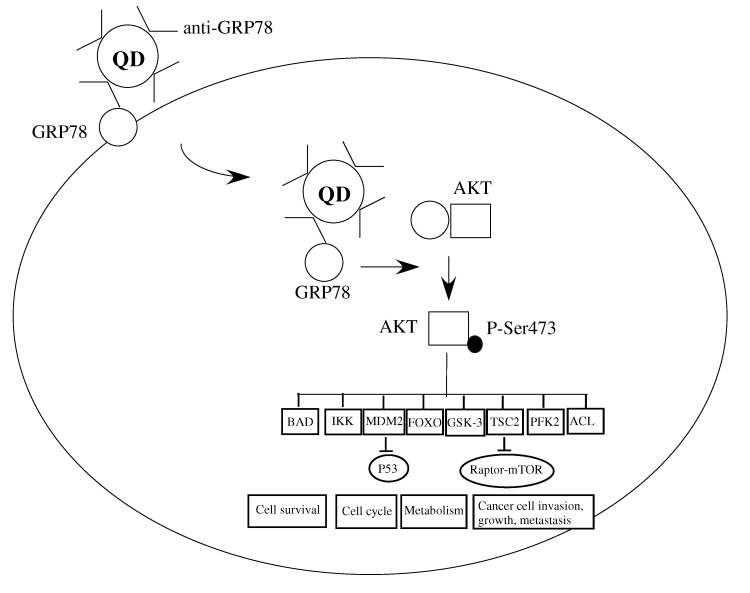
Schematic illustration of the Qdot-GRP78-scFv and GRP78 complex. The complex enters the intracellular space through GRP78 movement from cell into cytoplasm. The Qdot-GRP78-scFv:GRP78 complex prevents GRP78 binds to AKT, freeing AKT to be phosphorylated at Ser374, thereby mediating its downstream target processing including cancer cell invasion and growth.

Recently, down-regulation of GRP78 by SiRNA in different human cell lines, such as JAR (human choriocarcinoma cell) and HUVECs (human umbilical vein endothelial cells, have shown p-AKT upregulation [15]. p-AKT activation is well known to be involved in cell survival. But, as Yoeli-Lerner *et al*. pointed out p-AKT also blocked cancer invasion in MDA-MB-435, MDA-MB-231 and SUM-159-PT breast cancer cells [16]. Furthermore, activation of AKT has been found to suppress tumor invasion and metastasis in an *in vivo* transgenic model [17]. We propose the following mechanism for cancer growth inhibition by our Qdot-GRP78 antibody. The antibody binds cell surface GRP78 and is transported into the cytoplasma in the complex form. The GRP78:Qdot-GRP78 antibody complex prevents GRP78 binding to AKT, leading the AKT-Ser473 phosphorylation, which in turn, regulates a wide range of downstream events, including cancer cell invasion, growth and metastasis (Figure 5).

Recent rapid progresses in nanotechnology have had a great impact on cancer research and other clinical research [18]. Quantum dots have relatively long half life and stability [2], therefore conjugating QD with scFV could lead to scFv stabilisation. Most reports did not demonstrate the adverse effects of Qdots on cell viability, morphology and function over the duration of the experiments [2], other share suggested at a relatively high dose, Qdots may affect organ development [19]. Nevertheless the improved Qdot preparation will open up a new avenue for its therapeutic usage in medicine [20].

## 3. Experimental Section

### 3.1. scFv-GRP78-H19 Antibody Production

ScFvs in *E. coli* HB2151 were purified as soluble proteins from the periplasm of the bacteria by pelleting a 2 litre culture sample, resuspending the bacteria in 50 mL of PBS, followed by lysis and sonication. The cell debris were pelleted by centrifugation at 12,000 × g for 30 min at 4 °C. The supernatant, which contained the anti-GRP78 scFv protein, was purified using a Ni-TED spin column chromatography according to the manufacturer’s recommended conditions (Protino Ni-TED, Fisher Scientific, UK). The final elution was dialyzed against PBS using a Slid-A-lyzer mini dialysis unit (Thermo Scientific, UK). Protein concentration was measured using the Bio-Rad Protein Assay kit, Cat: 500-0006, Bio-Rad. UK. Protein purity was determined by SDS-PAGE and visualized by Coomassie Blue stain and was judged to be over 90% pure.

### 3.2. Quantum Dot Conjugates scFv-GRP78

ScFv-Grp78-H19 antibody (10) was conjugated with a Qdot-625 Antibody Conjugation Kits (Cat A10197, Invitrogen, UK) according to the manufacturer’s recommended conditions. Briefly, Qdots are activated with the heterobifunctional cross-linker 4-(maleimidomethyl)-1-cyclohexanecarboxylic acid *N*-hydroxysuccinimide ester (SMCC). At the same time, the antibody is reduced by DTT to expose free sulfhydryls. Then, activated Qdots are covalently coupled with reduced antibody and the reaction is quenched with β-mercaptoethanol. Conjugates were concentrated by ultrafiltration and purified by size exclusion chromatography. Dot blot and Western blot were used to characterise the specificity of the antibody. Immunohistochemistry was used to detect the presence of GRP78 in the breast cancer cells in culture cell. Furthermore, we have tested its function in nude mouse xenograft nude mouse model.

### 3.3. Cell Culture, Fluorescence Immunohistochemistry and Western Blot Analysis

MDA-MB-231/GFP human breast cancer cells were obtained from Cell Biolabs, Inc, CA, USA. Cells were grown in DMEM medium with 10% fetal calf serum in 37° incubator with 5% CO_2_. Immunofluorescence was used to visualize GRP78 protein in MDA-MB-231/GFP cells. Cells were grown on cover-slips in 6-well plates, washed with PBS twice, and fixed with PBS/4% paraformaldehyde for 30 min at room temperature. After fixation, the cells were stained with 10 nM Qot-GRP78 antibody overnight. The cells were washed with PBS and examined under a fluorescence microscope using a FITC filter. For studying antibody internalization, cells were incubated with 10 nM Qot-GRP78 antibody overnight and washed with PBS and directly examined under a fluorescence microscope. For 3D image reconstruction, the images were captured on a confocal microscope (Leica TCS SP, Germany) with Z stack at 0.8-μM and 3D reconstruction using the Leica 3D program. Western blot analysis was carried out as previous described [10]. An anit-GRP78 monoclonal antibody and Alexa fluor 488-labelled goat anti mouse IgG were from BD Bioscience, Oxford, UK. The anti-N20 GRP78 and an anti-α-tubulin antibody was from Insight Biotechnology, London, UK. Anti-phosphot-Akt (ser473) and anti-Akt (pan) were from the Cell Signaling Technology, Inc (Danvers, USA). *C*ells were grown to 60% confluence in chamber slides fixed with 4% paraformaldehyde in PBS for 10 min. The cells were then washed with PBS and permeabilized in PBS containing 0.1% Triton X-100 and 5% bovine serum albumin for 30 min. For detection of GRP78, the cells were stained with a 1:1,000 dilution of anti-GRP78 (Monoclonal antibody, BD) and a 1:500 dilution of anti-mouse Alexa488-conjugated secondary antibody (Invitrogen, UK). Cells were visualized on a confocal microscope (Leica TCS SP, Germany).

### 3.4. Preclinical Xenograft Nude Mouse Model

Young adult (6–8 weeks) Balb/c *nu/nu* mice from Harlan Laboratories were used for the xenograft MDA-MB-231/GFP human breast cancer cells. The care of experimental animals was in accordance with the University of Sheffield institutional guidelines. To produce subcutaneous tumors in Balb/c nude mice, suspensions of tumour cells in 1.6 mL of PBS were mixed with 0.4 mL of matrigel (BD Bioscience, UK). A total of 2 × 10^6^ cells were injected (with a 23-gauge needle) subcutaneously into the lateral flank proximal to the midline of the mouse. Each experiment used five female animals. When the tumor reached a volume of approximately 50–100 mm^3^ within a two to three week period, Qdot-625-scFv-GRP78 (80 μL containing 80 nM antibody) were intratumorally injected to the pre-established GFP labeled tumors. Tumor growth and antibody distribution can be monitored by visualization of multi-color fluorescence imaging (green representing tumor, red representing antibody) with a single excitation source (470 nm excitation filter) and single emission filter (515 nm viewing filter) (LightTools Research, Encinitas, CA). A total of three injections were carried out at weekly intervals. In the control group, only unlabeled nanobeads(80 nM) was injected. The mean diameter of tumors was measured with calipers once a week. Tumor volume was estimated using the following formula: tumor volume = length × width^2^ × 1/2. The statistical analysis was performed using a paired *student t*-test. A probability value of less than 5% (*p* < 0.05) was considered significant. Mice were sacrificed by cervical dislocation after seven weeks and tumors and some organs (spleen, kidney, heart, liver and lung) were removed and frozen for histology examination. Tissue collections were cryosectioned into 10-μm and imaged on the fluorescence microscope.

### 3.5. Immunohistochemistry of Anti-Grp78 scFv Antibody on Tissue Section

High-density paraffin-embedded breast cancer microarrays were purchased from Clinomics Biosciences (Pittsfield, MA, USA). Sections were dewaxed using routine dewaxing protocols. After blocking endogenous peroxidase activity with 3% H_2_O_2_ for 15 min, the antigen was retrieved using 1% SDS in TBS buffer (100 mM Tris pH 7.4, 138 mM NACl, 27 mM KCl). The sections were stained with scFv-Grp78-H19 at concentration of 5 μg/mL in PBSA solution (0.1% Triton X-100 in PBS) overnight. The second antibody was targeted against the c*-myc*-tag in the antibody (mouse MAb 9E10, Cambridge Research Biochemicals, Wilmington, DE, USA) and used at 1:1,000 dilution. A standard Vecta-stain ‘ABC kit’ (Vector Laboratories) was used with indirect avidin-biotin horseradish peroxidase staining.

### 3.6. Detection of Cellular Apoptosis

MDA-MB-231/GFP human breast cancer cells were grown to 60% confluence in chamber slides. Apoptosis and nuclear chromatin condensation was carried out with the Hoechst dye H33342 (20 μg mL^−1^) for 30 min, and nuclei were visualized using a Nikon Eclipase E400 fluorescence microscope with the Lucia image program. A minimum of 200 cells were counted in each sample and condensed or fragmented nuclei were expressed as a percentage of the total number of nuclei [10].

## 4. Conclusions

In summary, we report here that a Qdot-labelled anti-GRP78 scFv antibody can be used to detect GRP78 protein in both live and fixed cancer cells. Moreover we have demonstrated for the first time that Qdot-GRP78 scFv has biological tumoricidal activity and inhibits breast tumour growth in a xenograft *in vivo* model. The majority of previous studies using Qdots in biomedicine have been concerned with drug delivery, diagnostics, imaging and cell tracking. The therapeutic success of this study is potentially due to the ability of the labeled nanobead antibody to be internalised into the cancer cells through surface expression of the GRP78 protein.

## Supplementary Materials

Supplementary materials can be accessed at: http://www.mdpi.com/1420-3049/17/1/796/s1.

## References

[1] Rogach A.L. (2008). Semiconductor Nanocrystal Quantum Dots: Synthesis, Assembly, Spectroscopy, and Applications.

[2] Jaiswal J.K., Mattoussi H., Mauro J.M., Simon S.M. (2003). Long-term multiple color imaging of live cells using quantum dot bioconjugates. Nat. Biotechnol..

[3] Tada H., Higuchi H., Wanatabe T.M., Ohuchi N. (2007). *In vivo* real-time tracking of single quantum dots conjugated with monoclonal anti-HER2 antibody in tumors of mice. Cancer Res..

[4] Nelson A.L. (2010). Antibody fragments: Hope and hype. Mabs.

[5] Chen Y., Zhu X., Zhang X., Liu B., Huang L. (2010). Nanoparticles modified with tumor-targeting scFv deliver siRNA and miRNA for cancer therapy. Mol. Ther..

[6] Lee A.S. (2007). GRP78 induction in cancer: Therapeutic and prognostic implications. Cancer Res..

[7] Sato M., Yao V.J., Arap W., Pasqualini R. (2010). GRP78 signaling hub a receptor for targeted tumor therapy. Adv. Genet..

[8] Arap M.A., Lahdenranta J., Mintz P.J., Hajitou A., Sarkis A.S., Arap W., Pasqualini R. (2004). Cell surface expression of the stress response chaperone GRP78 enables tumor targeting by circulating ligands. Cancer Cell.

[9] Xu W., Liu L., Charles I.G., Moncada S. (2004). Nitric oxide induces coupling of mitochondrial signalling with the endoplasmic reticulum stress response. Nat. Cell. Biol..

[10] Liu L., Xu W. (2009). Targeting nitric oxide mediated upregulation of membrane-bound glucose regulated-protein 78 by subtractive single chain Variable Fragment (scFv) phage display. Am. J. Biomed. Sci..

[11] Daneshmand S., Quek M.L., Lin E., Lee C., Cote R.J., Hawes D., Cai J., Groshen S., Lieskovsky G., Skinner D.G., Lee A.S., Pinski J. (2007). Glucose-regulated protein GRP78 is up-regulated in prostate cancer and correlates with recurrence and survival. Hum. Pathol..

[12] Misra U.K., Mowery Y., Kaczowka S., Pizzo S.V. (2009). Ligation of cancer cell surface GRP78 with antibodies directed against its COOH-terminal domain up-regulates p53 activity and promotes apoptosis. Mol. Cancer Ther..

[13] Burikhanov R., Zhao Y., Goswami A., Qiu S., Schwarze S.R., Rangnekar V.M. (2009). The tumor suppressor Par-4 activates an extrinsic pathway for apoptosis. Cell.

[14] Kelber J.A., Panopoulos A.D., Shani G., Booker E.C., Belmonte J.C., Vale W.W., Gray P.C. (2009). Blockade of Cripto binding to cell surface GRP78 inhibits oncogenic Cripto signaling via MAPK/PI3K and Smad2/3 pathways. Oncogene.

[15] Yung H.W., Charnock-Jones D.S., Burton G.J. (2011). Regulation of AKT phosphorylation at Ser473 and Thr308 by endoplasmic reticulum stress modulates substrate specificity in a severity dependent manner. PLoS One.

[16] Yoeli-Lerner M., Yiu G.K., Rabinovitz I., Erhardt P., Jauliac S., Toker A. (2005). Akt blocks breast cancer cell motility and invasion through the transcription factor NFAT. Mol. Cell.

[17] Hutchinson J.N., Cardiff J., Jin R.D., Woodgett J.R., Muller W.J. (2004). Activation of Akt-1 (PKB-alpha) can accelerate ErbB-2-mediated mammary tumorigenesis but suppresses tumor invasion. Cancer Res..

[18] Loizidou M., Seifalian A.M. (2010). Nanotechnology and its applications in surgery. Br. J. Surg..

[19] Shiohara A., Hoshino A., Hanaki K., Suzuki K., Yamamoto K. (2004). On the cyto-toxicity caused by quantum dots. Microb. Immunol..

[20] Michalet X., Pinaud F.F., Bentolila L.A., Tsay J.M., Doose S., Li J.J., Sundaresan G., Wu A.M., Gambhir S.S., Weiss S. (2005). Quantum dots for live cells, *in vivo* imaging, and diagnostics. Science.

