# Supplementary material for: Quantum Dot- Conjugated Anti-GRP78 scFv Inhibits Cancer Growth in Mice
Source: Molecules. 2012 Jan 16;17(1):796–808. doi: 10.3390/molecules17010796 (PMC6268310; doi:10.3390/molecules17010796)
Supplement: Supplementary file 1 [file molecules-17-00796-s001.doc]

**Figure S1.** Representative images of the frozen cryostat mouse tissue sections at the end of 7 weeks intratumor injections of Qdot-GRP78. The frozen tissue sections were placed under a Nikon E400 fluorescence microscope, observed by either phase contrast or fluorescence setting (excitation filter 450–490 nm with emission filter 605/605 nm). Scale bar represents 20 µm.


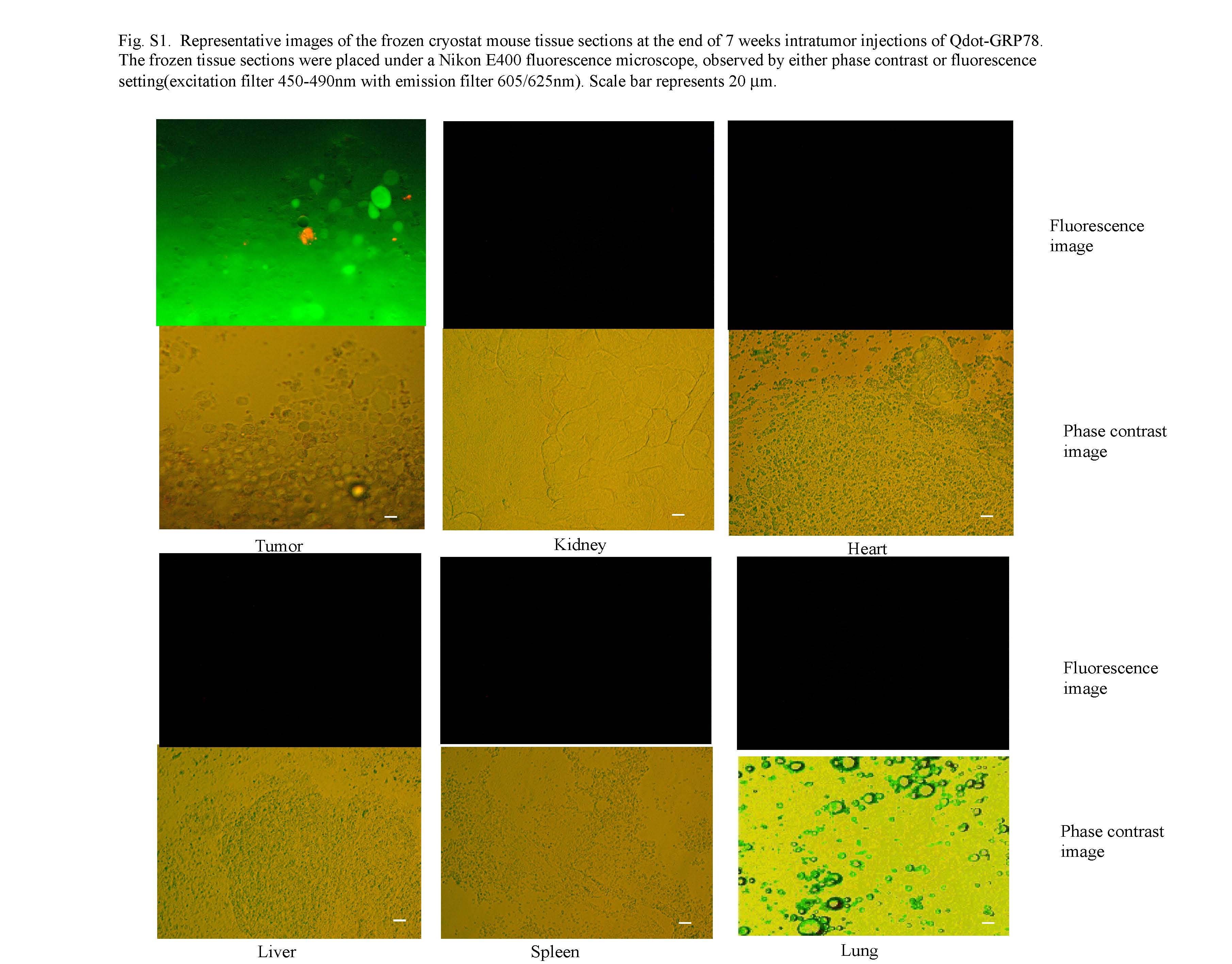


**Figure S2.** Immunohistochemistry results obtained from breast cancer samples in a tissue array stained with scFv-Grp78-H19. A. An immunohistochemical stain of a section from an breast adenocarcinoma (Tx N1 stage II, Clinomics Bioscience # 13498). Strong staining (brown) was observed on the membrane of the cancer cells(arrow). (scale bar represents 20 μm on paired samples). B. Control experiments omitting the primary (scFv-Grp78-H19). C. Representative images of the lymph nodes from a metastatic infiltrating adenocarcinoma (Clinomics Bioscience # 13712), showing strong staining with scFv-Grp78-H19. D. Benign specimen from the lymph node (Clinomics Bioscience # 13710), showing only weak staining (scale bar represents 20 μm on paired samples). Arrows indicate the membrane stain.
